# Understanding healthcare workers’ knowledge and attitudes regarding the coronavirus disease 2019 (COVID-19) vaccine: Overcoming gaps to improve public health response

**DOI:** 10.1017/ash.2022.335

**Published:** 2022-12-15

**Authors:** James Mo, Catherine J. Maples, Amanda Harris, Monica P. Meyer, Teena Chopra

**Affiliations:** 1 Wayne State University School of Medicine, Detroit, Michigan; 2 Division of Infectious Diseases, Department of Internal Medicine, Wayne State University School of Medicine, Detroit, Michigan

## Abstract

Vaccines serve as a major tool against the coronavirus disease 2019 (COVID-19) pandemic, but vaccine hesitancy remains a major concern in the United States. Healthcare workers (HCWs) strongly influence a patient’s decision to get vaccinated. We evaluated HCW knowledge and attitudes regarding the COVID-19 vaccine.

The novel severe acute respiratory coronavirus virus 2 (SARS-CoV-2) virus has caused >590 million cases of coronavirus disease 2019 (COVID-19) and 6.4 million deaths globally.^1^ Vaccines have been one of the greatest tools in public health and have contributed to the major decline in cases, hospitalizations, deaths, and healthcare-associated costs with vaccine-preventable diseases.^2^ A strong public health response aims to prevent disease, reduce disease transmission, and decrease the chance of developing new variants. To be successful at a community level, vaccine uptake must be high. However, vaccine hesitancy is a major barrier. In 2019, the World Health Organization ranked vaccine hesitancy as the tenth greatest threat to global health.^3^

To increase vaccine uptake, building public trust in the healthcare system is crucial. Healthcare workers (HCWs) remain the most trusted advisors of vaccine decisions among patients.^4^ Trust can be earned by increasing HCW vaccination rates and effectively educating HCWs on vaccine benefits. The participation of HCWs influences the public’s decision on whether to be vaccinated. Thus, identifying factors that limit HCWs’ ability to advise their patients on vaccines is important in reducing vaccine hesitancy and increasing vaccination rates. In this study, we evaluated HCW knowledge and attitudes regarding the COVID-19 vaccine.

## Methods

A cross-sectional survey using a validated self-administered questionnaire was generated and distributed through Qualtrics software to HCWs. This included physicians, resident physicians, medical students, medical assistants, and other HCWs. The survey was distributed through electronic mail, social media, and flyers. Responses were collected from August 2021 through February 2022. This study was approved by Wayne State University’s Institutional Review Board.

We used the 37-question voluntary and anonymous survey to collect information on respondents’ demographics, vaccination status, and knowledge and attitudes regarding vaccines including the COVID-19 vaccine. Responses were collected using a 5-point Likert scale ranging from “highly confident” to “highly not confident” or “strongly agree” to “strongly disagree.”

## Results

In total, 228 HCWs fully responded to the survey: 163 (71.5%) were women, 146 (64.0%) were white, and 165 (72.4%) were aged <40 years. In addition, 44 (19.3%) were physicians, 13 (5.7%) were resident physicians, 97 (42.5%) were medical students, 60 (26.3%) were medical assistants, and 14 (6.1%) were “other.” In total, 93% were up to date with the recommended adult vaccine schedule. Among all respondents, 211 (92.5%) had received the COVID-19 vaccine and 2 (0.9%) planned to receive it.

We evaluated the survey data regarding HCW knowledge and attitudes regarding vaccines (Table 1). Only 64% of respondents agreed that the only way to eradicate an infectious disease was through vaccination. Also, 3% agreed that vaccines increase the risk of autism or that the most common adverse reaction to vaccines is not an injection site reaction, and 9% were unsure. Furthermore, 5% of respondents did not believe that vaccine hesitancy is a national problem, and 66% stated that they trusted the government and public health policy interventions in preventing disease. However, 61% also believed that people should continue to wear masks regardless of recommendations from the Center for Disease Control and Prevention (CDC).

**Table 1.** Healthcare Workers’ Knowledge and Attitudes Regarding Vaccines and Public Health Response (n = 228)

Survey Item	Strongly Agree or Highly Confident, No. (%)	Agree or Somewhat Confident, No. (%)	Neutral or Unsure, No. (%)	Disagree or Somewhat Not Confident, No. (%)	Strongly Disagree or Highly Not Confident, No. (%)
**Knowledge and attitudes regarding vaccines and public health response**					
Vaccination is the only way to eradicate infectious disease.	55 (24.1)	90 (39.5)	45 (19.7)	25 (8.3)	13 (5.7)
Vaccines increase the risk of autism.	0 (0.0)	4 (1.8)	11 (4.8)	39 (17.1)	174 (77.2)
The most common adverse reaction to vaccination is an injection site reaction.	122 (53.5)	93 (40.8)	10 (4.4)	3 (1.3)	0 (0.0)
Vaccine hesitancy is a national problem.	134 (58.8)	70 (30.7)	13 (5.7)	8 (3.5)	3 (1.3)
Trust the government and public health policy interventions to prevent disease.	72 (31.6)	78 (34.2)	49 (21.5)	18 (7.9)	11 (4.8)
People should continue to wear masks regardless of Center for Disease Control and Prevention (CDC) recommendations.	67 (29.4)	73 (32.0)	46 (20.2)	26 (11.4)	16 (7.0)
Religion and moral values influence an individual’s willingness to be vaccinated.	58 (25.4)	112 (49.1)	35 (15.4)	19 (8.3)	4 (1.8)
**Confidence levels regarding the COVID-19 vaccine**					
Efficacy	114 (50.0)	73 (32.0)	12 (5.3)	14 (6.1)	15 (6.6)
Safety	140 (61.4)	47 (20.6)	12 (5.3)	13 (5.7)	16 (7.0)
Duration of immunity	32 (14.0)	107 (46.9)	47 (20.6)	19 (8.3)	23 (10.1)
Need for a booster shot after initial vaccination	89 (39.0)	74 (32.5)	30 (13.2)	14 (6.1)	21 (9.2)
**Confidence levels regarding various information sources**					
CDC	73 (32.0)	81 (35.5)	29 (12.7)	20 (8.8)	25 (11.0)
State and local health departments	66 (29.0)	86 (37.7)	37 (16.2)	19 (8.3)	20 (8.8)
News media	8 (3.5)	39 (17.1)	49 (21.5)	72 (31.6)	60 (26.3)
Social media	2 (0.9)	14 (6.1)	27 (11.8)	67 (29.4)	118 (51.8)
Friends and family	3 (1.3)	38 (16.7)	47 (20.6)	75 (32.9)	65 (28.5)
Themselves advising a vaccine hesitant patient on the risks and benefits of immunization	82 (36.0)	109 (47.8)	27 (11.8)	9 (4.0)	1 (0.4)

In this study, we also evaluated HCW attitudes regarding the COVID-19 vaccine. Among respondents, 82% were confident in both the efficacy and safety of the vaccine. Furthermore, 61% were confident in the duration of immunity provided by the vaccine, and 72% also believed that booster shots were needed after initial vaccination.

Finally, attitudes regarding various information sources throughout the COVID-19 pandemic were surveyed. Only 68% and 67% of respondents were confident in the accuracy of information reported by the CDC and state and local health departments, respectively. Confidence was even lower in the accuracy of information reported by the news media (21%), social media (7%), and friends and family (18%). These data may be related to our additional finding that 76% believed that religion and moral values influence an individual’s willingness to be vaccinated. In contrast, 84% of respondents were confident in themselves being able to advise vaccine-hesitant patients on immunizations.

## Discussion

These findings identify a critical gap in HCW knowledge and attitudes regarding COVID-19 vaccinations. Despite 94% of HCWs having received or planned to receive the COVID-19 vaccine, many were not confident in the efficacy and safety of the vaccine. Some were also unaware of vaccine side effects. This finding is concerning because most HCWs were confident in advising vaccine-hesitant patients, which could result in HCWs disseminating misinformation to their patients.

Misinformation is prevalent despite the evidence-based data provided by government officials on the COVID-19 vaccine. One reason may be that HCWs do not actually have confidence in government officials despite stating they do. This disparity is evident by their stated disregard for the CDC mask policy. Distrust began early in the pandemic due to inconsistent and differing guidelines over proper use of personal protective equipment. This inconsistency led to confusion and avoidable errors by HCWs and increased contamination and infection.^5^ In addition, HCWs were required to receive the COVID-19 vaccine by their institutions regardless of their knowledge of or confidence in the vaccine. Finally, there was concern over the mRNA technology used to develop the COVID-19 vaccines. An analysis found that the novelty of the mRNA technology reduced the odds of accepting the vaccine by 14.2%. The same study also found that social conformity can reduce vaccine hesitancy.^6^ Therefore, increasing HCW knowledge and confidence in the vaccine is necessary for them to be able to better recommend the COVID-19 vaccine to their patients.

Multiple strategies can be employed to increase vaccine acceptance in the public. Continued medical education (CME) is needed to address any confusion HCWs have over the COVID-19 vaccine. Physician participation in previous CME on the influenza and pneumococcal vaccines led to increased adult vaccination rate in patients.^7^ The information gaps identified by this study can be used to guide future CME. One topic that needs clarification is the need for booster shots despite confidence in the duration of the COVID-19 vaccine. Next, HCWs need to be placed on the frontlines of the media to be able to better disseminate accurate information. HCWs were correct to believe personal identity and beliefs play a role in one’s decision to get vaccinated. Identified factors include race, religion, and political identification.^8^ These factors can affect the accuracy of information any individual decides to broadcast through the news and social media. This is also true of one-on-one interactions. HCWs need to be in communities directly interacting with community members. In one study, following one-on-one patient education sessions, medication adherence increased to 94.4% compared to 89.9%.^9^ The same can be done for the COVID-19 vaccine through initiatives such as vaccine clinics and door-to-door campaigns.

This study had several limitations. Most respondents were young, white, and female. Thus, more diverse populations need to be surveyed. In addition, because of the method of distribution and anonymity, respondents’ places of employment, affiliations, and vaccine requirements were unknown, which could have affected the high COVID-19 vaccination rate. Finally, this survey was distributed before recommendations for second booster shots were available, potentially changing responses. The present study provides information on HCW knowledge and attitudes regarding the COVID-19 vaccine. These findings will help address vaccine hesitancy in the United States and bolster the public health response to the COVID-19 pandemic.
